# Effect of changing treatment to high-flux hemodialysis (HFHD) on mortality in patients with long-term low flux hemodialysis (LFHD): a propensity score matched cohort study

**DOI:** 10.1186/s12882-020-02145-5

**Published:** 2020-11-16

**Authors:** Shuxin Liu, Hong Liu, Zhihong Wang, Lanbo Teng, Cui Dong, Tingting Gui, Yu Zhang

**Affiliations:** Department of Nephrology, Dalian municipal central hospital, No. 826, Southwest Road, Dalian, Liaoning Province 116033 PR China

**Keywords:** High-flux hemodialysis, Low-flux hemodialysis, End-stage renal disease, Prognosis, Propensity score matched cohort study

## Abstract

**Background:**

The purpose of this study was to explore the effect of changing treatment to high-flux hemodialysis (HFHD) on mortality rate in patients with long-term low flux hemodialysis (LFHD).

**Methods:**

The patients with end-stage renal disease (ESRD) who underwent LFHD with dialysis age more than 36 months and stable condition in our hospital before December 31, 2014 were included in this study. They were divided into control group and observation group. Propensity score matched method was used to select patients in the control group. The hemodialysis was performed 3 times a week for 4 h. The deadline for follow-up is December 31, 2018. End-point event is all-cause death. The survival rates of the two groups were compared and multivariate Cox regression analysis was carried out.

**Results:**

K-M survival analysis showed that the 1-year, 2-year, 3-year and 4-year survival rates of HFHD group were 98, 96, 96 and 96%, respectively. The 1-year, 2-year, 3-year and 4-year survival rates of LFHD group were 95, 85, 80 and 78%, respectively. Log-rank test showed that the survival rate of HFHD group was significantly higher than that of LFHD group (x^2^= 7.278, *P* = 0.007). Multivariate Cox regression analysis showed that male, age, hemoglobin and low-throughput dialysis were independent predictors of death (*P* < 0.05). Compared with LFHD, HFHD can significantly reduce the mortality risk ratio of patients, as high as 86%.

**Conclusion:**

The prognosis of patients with ESRD who performed long-term LFHD can be significantly improved after changing to HFHD.

## Background

Despite the continuous development of blood purification technology, the annual mortality and complications of patients with end-stage renal disease (ESRD) are still high. The annual mortality rate of patients who perform maintenance hemodialysis is about 18%, the average number of hospitalization is 1.9, and the average length of hospitalization is 14.0 days, of whom cardiovascular disease is the main cause of death [1]. Compared with low-flux hemodialysis (LFHD), high-flux hemodialysis (HFHD) can more effectively remove intermediate molecular uremic toxins with molecular weights of 5000–15,000 Da, such as β2-MG etc. [2]. However, neither the MPO study [3] nor the HEMO study [4] have been able to obtain the result of HFHD to improve the prognosis of patients. It is suggested that there are many factors that affect the prognosis of patients with ESRD, such as volume, blood pressure, anemia, inflammation, and mineral-bone metabolism disorders. Clearance of uremic toxins may be only one of the influencing factors.

However, further subgroup analysis of the HEMO study showed that patients with long dialysis age (more than 3.7 years) before randomization stratification significantly benefited from HFHD treatment in comparison with LFHD [5]. In above study, the risk of all-cause death decreased by 32%, and the risk of cardiovascular disease death decreased by 37%, suggesting that dialysis time is an important factor affecting whether HFHD and LFHD will benefit. In other words, HFHD may be beneficial to the prognosis of patients with long-term LFHD. At present, few research has done on it. Therefore, we investigated the effect of HFHD on the prognosis of patients who performed long-term LFHD, and laid the foundation for further clarifying the treatment benefits of HFHD.

### Patients and methods

#### Patients

This study was approved by the ethics committee of Dalian Central Hospital. All patients signed informed consent. A total of 171 patients with ESRD who underwent LFHD with dialysis age more than 36 months and stable condition in our hospital before December 31, 2014 were included in this study. They were divided into control group (*n* = 57) and observation group (*n* = 114). The patients who voluntarily changed to perform HFHD were the observation group. The patients with LFHD who matched with age, gender, primary disease, dialysis age, hemoglobin, albumin, blood phosphorus, KT/V, cardiovascular and cerebrovascular complications of patients in observation group were selected as the control group. Inclusion criteria: (1) Patients undergoing LFHD in our hospital before November 30, 2014. (2) Patients who voluntarily changed to perform HFHD between December 1, 2014 and December 31, 2014 were enrolled in the observation group. (3) Patients over 18 years old. (4) Dialysis age more than 36 months. (5) Patients with stable condition. Exclusion criteria: (1) Patients who had tumor. (2) Patients with myocardial infarction within 3 months. (3) Patients with New York Heart Association (NYHA) heart function grade III and above. (4) Patients who quit high flux dialysis after admission.

#### Therapeutic schedule

The patients in the observation group were performed with HFHD, and the patients in the control group were performed with LFHD for follow-up treatment. Dialysis treatment was performed with double reverse osmosis water (Raul water machine) and standard bicarbonate, 3 times a week, 4 h each time. The definition of low flux dialyzer is that the clearance of β 2-mg is 10 ml/min. The definition of high flux dialyzer is that the clearance of β 2-mg is 20 ml/min.

#### Observation indexes

The end point was all cause death. The deadline for follow-up was December 31, 2018. Follow up time was recorded in months. Deletion value was defined as patients who are still alive or lost to follow-up by December 31, 2018.

### Statistical analysis

Statistical analysis was made by software SPSS22.0 (International Business Machines, corp., Armonk, NY, USA). The measurement data is expressed by mean ± standard deviation (SD). Measurement data with normal distribution were compared with t test, and those with non-normal distribution were compared with U test. Chi square test was used to compare the counting data. Kaplan meter analysis was used for survival analysis, and log rank test was used for survival comparison between the two groups. Univariate factor Cox regression analysis was used for risk assessment. Multivariate Cox regression was used for independence analysis. Differences were considered statistically significant when *p* < 0.05.

## Results

### General clinical features

A total of 57 eligible patients were transferred from LFHD to HFHD. One hundred fourteen patients in the control group were generated using the 1: 2 propensity score ratio analysis. There were 114 males and 57 females with an average age of 49.5 ± 11.8 years. The primary diseases were chronic glomerulonephritis in 96 patients, diabetic nephropathy in 18 patients, hypertension in 42 patients, and other diseases in 15 patients. There were no significant differences in sex, age, distribution of primary disease, age of dialysis, hemoglobin, albumin, blood phosphorus, KT/V and incidence of cardiovascular complications between the two groups (*p* > 0.05, respectively) (Table 1).

**Table 1:** General clinical features

Groups	HFHD group	LFHD group	*p*
Gender (male/female)	76/38	38/19	1.000
Age (years)	50.1 ± 9.8	49.3 ± 12.7	0.055
Primary disease			0.836
Chronic glomerulonephritis	33	63	
diabetic nephropathy	6	12	
Hypertensive renal damage	13	29	
Other diseases	5	10	
Dialysis age (month)	83.2 ± 30.4	79.2 ± 45.3	0.243
Laboratory examination			
Hemoglobin (g/l)	112.79 ± 15.61	112.99 ± 13.86	0.973
Albumin (g/dl)	41.32 ± 2.05	41.35 ± 2.33	0.196
Blood phosphorus (mmol/l)	2.16 ± 0.54	2.14 ± 0.58	0.798
KT/V	1.35 ± 0.25	1.36 ± 0.22	0.813
Cardiovascular complications	4	2	1.00

### Survival analysis

The median follow-up time was 42.0 (2–48) months. In HFHD group, 2 patients died, 2 patients underwent renal transplantation, 5 patients lost follow-up; in IFHD group, 22 patients died, 1 patient underwent renal transplantation, 10 patients lost follow-up. A total of 24 patients had terminal events. The causes of death were cardiovascular disease in 15 patients (62.5%), stroke in 6 patients (25.0%), infection in 2 patients (8.3%), and tumor in 1 patient (4.2%). The 1-year, 2-year, 3-year and 4-year survival rates of HFHD group were 98, 96, 96 and 96%, respectively. The 1-year, 2-year, 3-year and 4-year survival rates of lfhd group were 95, 85, 80 and 78%, respectively.

**Fig. 1:**
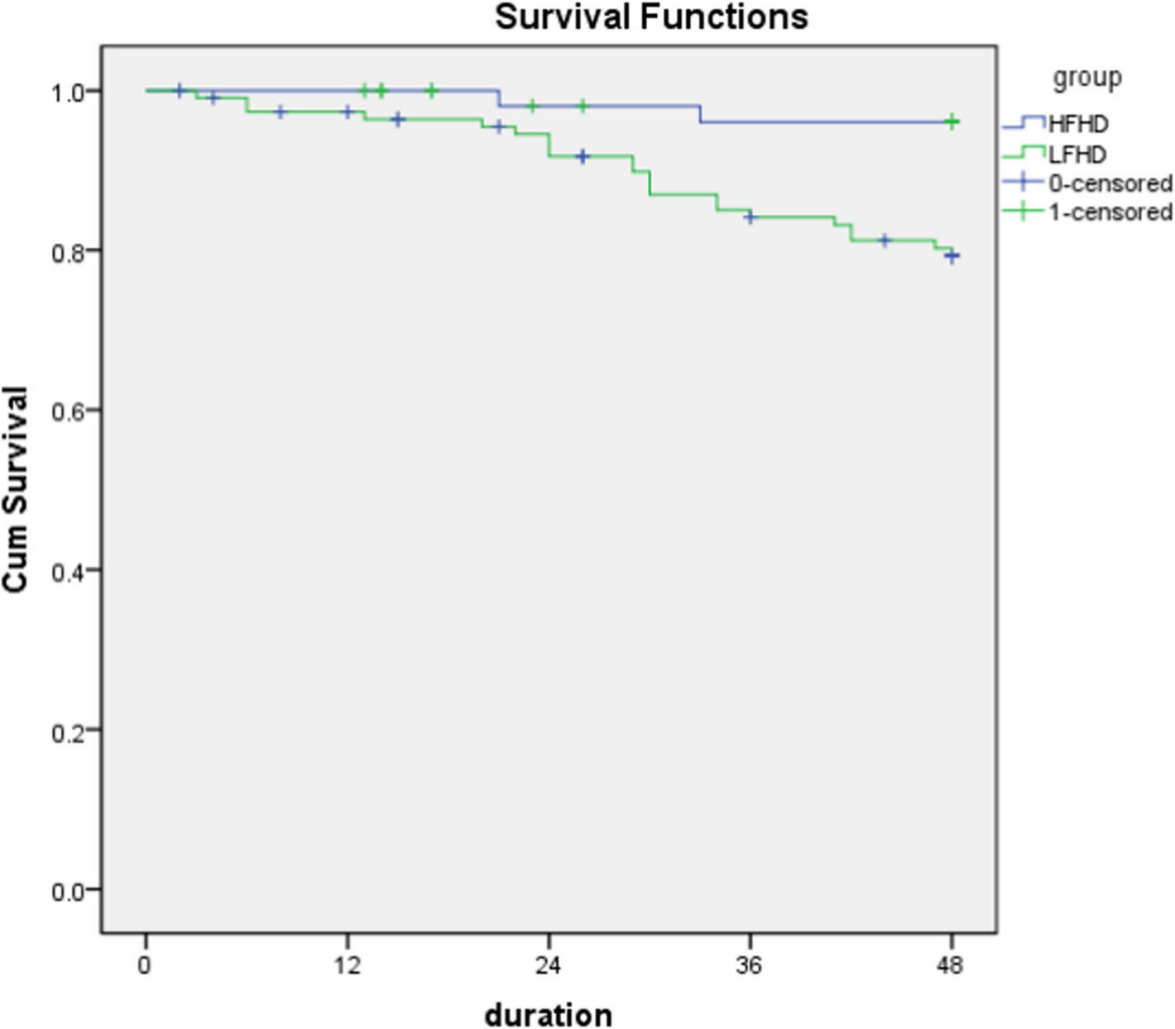
K-M survival analysis of patients between the two groups

Survival differences between the two groups was compared by using K-M survival analysis. Log-rank test showed that the survival rate of HFHD group was significantly higher than that of LFHD group (× 2 = 7.278, *p* = 0.007) (Fig. 1).

### Univariate cox regression analysis

Univariate Cox regression analysis was used to analyze the effect of various factors on death. It was found that gender, age, hemoglobin and HFHD were all statistically significant (*p* < 0.1) (Table 2).

**Table 2:** Univariate Cox regression analysis

Factors	*p* value	HR	95%CI for HR	
			Lower	Upper
Female	0.070	0.279	0.070	1.112
Age	0.001	1.095	1.047	1.145
Dialysis age	0.240	1.006	0.996	1.017
Hemoglobin	0.006	0.959	0.930	0.988
Albumin	0.355	1.091	0.907	1.314
Blood phosphorus	0.737	1.142	0.526	2.479
KT/V	0.166	0.186	0.017	2.005
Cardiovascular complications	0.129	2.817	0.739	10.735
High flux hemodialysis	0.010	0.138	0.030	0.628

### Multivariate cox regression analysis

The above factors are taken into the multivariate Cox regression analysis to analyze whether these factors predict the independence of death. It was found that male, age, hemoglobin and -LFHD were independent predictors of death (*p* < 0.05) (Table 3). Compared with LFHD, HFHD can significantly reduce the mortality risk ratio of patients, as high as 86%.

**Table 3:** Multivariate Cox regression analysis

Factors	*p* value	HR	95%CI for HR	
			Lower	Upper
Female	0.011	0.204	0.060	0.697
Age (years)	0.001	1.095	1.055	1.138
Hemoglobin (g / L)	0.002	0.955	0.928	0.984
HFHD	0.009	0.142	0.033	0.617

## Discussion

We conducted a retrospective, propensity score matching analysis cohort study. The results showed that the prognosis of patients with ESRD after long-term LFHD could be significantly improved after changing to HFHD.

HFHD has the advantages of eliminating medium molecular toxins and high cost performance, so it is more and more widely used in clinical practice. However, since MPO and HEMO studies, it has been debated whether HFHD can improve the survival and prognosis of patients compared with traditional LFHD. Most of the early clinical studies are similar to the results of HEMO and MPO studies, suggesting that there is no significant difference between HFHD and LFHD in all-cause death of patients with ESRD. However, recent meta-analysis and clinical studies have drawn different conclusions. A meta-analysis of 8 high-quality clinical studies involving 4967 patients with ESRD (2416 in the HFHD group and 2551 in the LFHD group) showed that all-cause mortality was significantly lower in the HFHD group than in the LFHD group (OR = 0.704, 95% CI = 0.533–0.929, *p* = 0.013), and the cardiovascular mortality in the HFHD group was significantly lower than that in the LFHD group (OR = 0.731, 95% CI = 0.616–0.866, *p* < 0.001), which suggested that patients with ESRD should be treated with HFHD [6]. The results of another meta-analysis are similar to it., in which a total of 4412 ESRD patients were included in 7 items. It was found that HFHD could reduce all-cause mortality and cardiovascular disease mortality by 25% compared with lfhd, but had no significant effect on infection mortality [7]. In a multi-center prospective observational cohort study in Korea, 1165 newly diagnosed ESRD patients and 1641 ordinary hemodialysis patients (dialysis time > 3 months) were enrolled. The dialysis characteristics and health measurement were conducted every 6 months until the end of the follow-up period. After a follow-up of 24 months, it was found that HFHD significantly reduced all-cause mortality (or = 0.606, 95% CI = 0.416–0.885, *P* = 0.009) in comparison with LFHD in patients with maintenance hemodialysis [8]. It was considered that HFHD reduced mortality. These results suggest that HFHD may improve the survival rate of patients.

In China, with the continuous popularity of HFHD, more and more patients who had long-term LFHD choose HFHD, so the prognosis of these patients is more and more concerned. At present, there is no prospective, randomized, multicenter study to confirm whether it can improve the prognosis of patients. HFHD technology was only carried out in our center at the end of 2014. Before, all patients had been treated with LFHD. Although the charges for the two were the same and doctors made positive recommendations, only some patients received HFHD treatment at the beginning. Most patients were worried about the treatment effect and did not choose HFHD treatment. Among the patients selected for HFHD treatment, the patients with dialysis age of 36 months or more were selected as the observation group of this study. Subgroup analysis of the HEMO study showed that compared with LFD, patients with long dialysis age (> 3.7 years) before randomization benefited significantly from HFD, including a 32% reduction in all-cause mortality and a 37% reduction in cardiovascular death risk. It is suggested that dialysis age is an important factor affecting the benefit of HFD or LFD. Therefore, we want to verify whether this conclusion is applicable to Chinese patients who underwent dialysis.

In this study, patients who had dialysis over 36 months and voluntarily changed to HFHD were enrolled in observation group. The patients who were performed with LFHD were selected as the control group by propensity matching analysis. The median follow-up time was 42 months. The results of this study showed that the risk of death was six times higher in the continuous LFHD group than that in the HFHD group. In other words, HFHD can significantly improve the prognosis of patients with ESRD who were performed with long-term LFHD. In terms of mechanism, the levels of molecular uremic toxins, such as β 2-microglobulin and oxidative stress, were higher in patients with LFHD. Compared with LFHD, LFHD can more effectively remove β2-microglobulin [9] and improve oxidative stress [10]. The results of HEMO study showed that β2-microglobulin level was a predictor of death in patients with maintenance hemodialysis, so reducing β 2-microglobulin level may reduce the risk of death [11]. In addition, FGF-23 is a risk factor for cardiovascular events and all-cause death in patients with ESRD. Compared with IFHD treatment, HFHD treatment can stabilize or even reduce the level of FGF-23 [12, 13], which may be one of the possible mechanisms for improving the prognosis of these patients. Recent metabonomics studies also showed that HFHD treatment was more effective than LFHD treatment in eliminating guanidine, hippuric acid and other 11 uremic toxins, most of which were related to inflammation, vascular endothelial function and other pathways [14].

In addition to the change of treatment mode, the study found that men, age and hemoglobin were also independent risk factors for the prognosis of patients with ESRD. There was no gender difference in most studies on the prognosis of patients with ESRD. Age itself is an important factor affecting the prognosis of ESRD, and the mortality increases with age. It has been pointed out that for every 10-year increase in age, the mortality rate of ESRD patients increases 1.2–2.1 times [15]. Low or high hemoglobin levels will affect the prognosis of ESRD patients. Current guidelines recommend that the hemoglobin level of patients with maintenance hemodialysis should be maintained at 110-120 g/L as the best level.

There were some advantages of this study. Although it is not a randomized controlled study, the propensity matching analysis method was used to match many other clinical factors affecting ESRD patients’ prognosis in addition to treatment mode. In addition, the follow-up period was longer. Furthermore, this study was in line with China’s national conditions and current treatment status. There were also some disadvantages of this study. We did not pay attention to factors such as education levels and family income of patients in the two groups that may affect treatment choices. In addition, the quality of life of the two groups was not evaluated. Moreover, evaluations and studies of therapeutic economics were not performed.

## Conclusion

In conclusion, the results of this study showed that the change of patients with ESRD with long-range LFHD to HFHD treatment can significantly improve the prognosis of patients, laying a foundation for clinical research to further popularize HFHD in the clinic. However, this also needs to be confirmed by large-scale, prospective, multicenter clinical studies.

## Data Availability

Not applicable.
